# Antibiotic prescribing for children for acute conditions in public primary care clinics in Singapore: a retrospective cohort database study

**DOI:** 10.1017/ash.2025.157

**Published:** 2025-09-03

**Authors:** Sky Wei Chee Koh, Vivien Min Er Lee, Si Hui Low, Anna Szuecs, Victor Weng Keong Loh, Meena Sundram, Linus Kee Loon Chua, José M. Valderas, Li Yang Hsu

**Affiliations:** 1 Division of Family Medicine, Yong Loo Lin School of MedicineNational University of Singapore, Singapore 119228, Singapore; 2 National University PolyclinicsNational University Health System, Singapore 609606, Singapore; 3 Saw Swee Hock School of Public HealthNational University of Singapore, Singapore 117549, Singapore

## Abstract

**Objectives:** Data on primary care antibiotic prescription practices for children in Southeast Asia, which are essential for policy, quality improvement and patient safety, are lacking. We aimed to describe this gap and to benchmark prescription practices against international standards. **Methods:** Antibiotic prescriptions for children (age <18 years) who visited six public primary care clinics in Singapore between 2018 and 2021 were extracted and categorized according to the World Health Organization Access, Watch, Reserve (WHO AWaRe) classification. Quality indicators from the European Surveillance of Antimicrobial Consumption Network (ESAC- Net) and National Institute for Health and Care Excellence (NICE) guidelines were used as a measure of appropriateness of antibiotic prescribing. Descriptive statistics and T-test was used to compare prescription rates pre- and post-COVID-19 pandemic. **Results:** 19,325 and 20,692 oral and topical antibiotics were prescribed for 831,669 visits, with a prescription rate of 2.3% and 2.5% respectively. Mean antibiotic prescriptions fell significantly post-pandemic (2020–2021), compared to pre-pandemic numbers (1062.8 to 604.5 prescriptions per month) (*p* <0.001). The majority (95.8%) of prescriptions belonged to the Access group. Watch group antibiotics constituted 6.1% of the total antibiotics prescribed for respiratory conditions (n = 562). While prescriptions were low (4.1%) and well within EASC-Net quality indicator limits of 0-20% for respiratory infections, prescriptions for otitis media were significantly high (56.6%). Approximately 1 in 2 children received antibiotics as recommended by NICE guidelines for both respiratory infections (n=4,622, 51.5%) and otitis media (n=204, 51.8%). **Conclusions:** Primary care antibiotic prescriptions for children in Singapore decreased post- COVID-19. However, high rates of otitis media prescriptions and only 50% appropriateness for respiratory infections and otitis media emphasize the need for targeted improvements in these areas.

